# Plastid Phosphatidylglycerol Homeostasis Is Required for Plant Growth and Metabolism in *Arabidopsis thaliana*

**DOI:** 10.3390/metabo13030318

**Published:** 2023-02-21

**Authors:** Mingjie Chen, Shiya Wang, Yi Zhang, Dongsheng Fang, Jay J. Thelen

**Affiliations:** 1 College of Life Sciences, Xinyang Normal University, Xinyang 464000, China; 2 Christopher S. Bond Life Science Center, Interdisciplinary Plant Group, Division of Biochemistry, University of Missouri, Columbia, MO 65203, USA; 3 School of Life Sciences, Nanchang University, Nanchang 330031, China

**Keywords:** *Arabidopsis thaliana*, *FATTY ACID DESATURASE 4*, lipidomics, phosphatidylglycerol, *trans*-double bond, polar lipids, growth rate, photoassimilate, export, chloroplast

## Abstract

A unique feature of plastid phosphatidylglycerol (PG) is a *trans*-double bond specifically at the *sn*-2 position of 16C fatty acid (16:1t- PG), which is catalyzed by FATTY ACID DESATURASE 4 (FAD4). To offer additional insights about the in vivo roles of FAD4 and its product 16:1t-PG, *FAD4* overexpression lines (*OX-FAD4s*) were generated in *Arabidopsis thaliana* Columbia ecotype. When grown under continuous light condition, the *fad4-2* and *OX-FAD4s* plants exhibited higher growth rates compared to WT control. Total lipids were isolated from *Col*, *fad4-2*, and *OX-FAD4_2* plants, and polar lipids quantified by lipidomic profiling. We found that disrupting *FAD4* expression altered prokaryotic and eukaryotic PG content and composition. Prokaryotic and eukaryotic monogalactosyl diacylglycerol (MGDG) was up-regulated in *OX-FAD4* plants but not in *fad4-2* mutant. We propose that 16:1t-PG homeostasis in plastid envelope membranes may coordinate plant growth and stress response by restricting photoassimilate export from the chloroplast.

## 1. Introduction

Plant fatty acids (FAs) are mainly synthesized de novo in the plastid stroma [1], then exported into the cytoplasm. According to acyl-ACP synthesis rates and specificity of thioesterases in *Arabidopsis* plastids, oleic acid (18:1) is the major FA exported from the plastid, followed by palmitic acid (16:0) and trace amounts of stearic acid (18:0) [2,3]. Plastid membrane lipids are synthesized by both the prokaryotic and eukaryotic pathways [4]. The prokaryotic pathway assembles the synthesized FAs de novo in the plastid; the eukaryotic pathway assembles the plastid-exported FAs into lipids within the endoplasmic reticulum (ER). Part of the ER-assembled lipids are trafficked back to the plastid, where they are converted to monogalactosyldiacylglycerol (MGDG), digalactosyldiacylglycerol (DGDG), and sulfoquinovosyldiacylglycerol (SQDG). Due to the difference in substrate specificities of acyltransferases at the ER and the plastid envelope [5], glycerolipids originating from the prokaryotic pathway have a 16-carbon acyl chain at the *sn*-2 position of the glycerol backbone, while glycerolipids assembled on the ER carry an 18-carbon acyl chain at the same position [6].

**Figure 1:**
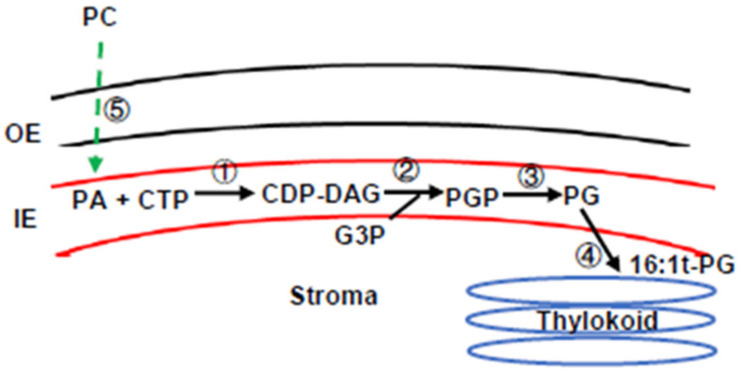
A diagram presentation of plastid PG synthesis in *Arabidopsis*. OE, chloroplast outer envelope mem-brane; IE, chloroplast inner envelope membrane; PA, phosphatidic acid; CTP, cytidine trios phosphate; CDP-DAG, cytidine diphosphate-diacylglycerol; G3P, glycerol-3-phosphate; PGP, phosphatidylglycerol phosphate; PG, phosphatidylglycerol; ①, CDP-DAG synthase; ②, PGP synthase 1; ③, PGP phosphatase 1; ④, FATTY ACID DESATURASE 4; ⑤, TGD complex.

In plant cells, plastid phosphatidylglycerol (PG) is the major phospholipid of the plastid envelope and thylakoid membranes and is also a minor component in other cellular membranes [7]. The plastid, ER, and mitochondria organelles are each capable of synthesizing PG from phosphatidic acid (PA) [8,9]. The plastid pathway for PG synthesis is located in the inner envelope (IE) membrane [10] (Figure 1). CDP-DAG synthase transfers the cytidylyl group from CTP to PA to form CDP-DAG [11]. PGP synthase 1 (PGP1) converts CDP-DAG and glycerol-3-phosphate (G3P) into phosphatidylglycerol phosphate (PGP) [9], then plastid PGP phosphatase 1 (PGPP1) converts PGP into PG [8]. The plant ER also possesses all enzyme activities for de novo PG synthesis [12], and PGP2 could be responsible for PG synthesis in ER [13]. The PC synthesized in ER could be transported into plastid inner envelope membrane through TGD complex, then converted into PG. Mitochondrial PG synthesis is mediated by a PGP1 isoform that targets to the mitochondria [14]. In addition, ER-assembled PG could be imported into the mitochondria to support cardiolipin synthesis in the inner mitochondria membrane [13,14]. Thus, PGP2 shows functional redundancy with the mitochondria PGP1 isoform even though they locate to different subcellular compartments.

A unique feature of plastid PG is a *trans*-double bond at the *sn*-2 position of the 16C fatty acid (16:1t- PG), introduced by FAD4 using PG (18:x/16:0) substrates but not PA or PGP as substrates [15,16]. FAD4 is a thylakoid-associated protein facing the stromal side [17] (Figure 1), and chloroplast PG is produced at the IE membrane [9,13,17]. Thus, plastid PG synthesis and desaturation are spatially separated. Plastid PG has been implicated in multiple functions, including chloroplast protein import [18], stabilization of PSI trimers [19], PSII core dimers [20], and light-harvest complex II. A leaky mutation for plastid PGP1 showed a reduction in total PG content of ~30%, leading to a pale green leaf color and impaired photosynthetic light energy utilization [9]. Complete knockout of the PGP1 gene leads to the abolishment of chloroplasts and loss of photoautotrophy [14]. In the *sqd2pgp1-1* double mutant, the fraction of total anionic lipids (PG + SQDG) was reduced by about one-third and accompanied by multiple defects, including a pale-yellow leaf color, compromised photoautotrophic growth, reduced numbers of mesophyll cells, and an altered chloroplast ultrastructure. Thus, the authors concluded that anionic lipids PG and SQDG play critical roles in the proper structure and function of photosynthetic membranes for seed plants [21]. Unlike *PGP1* knockout plants [14], *FAD4* abolishment showed no significant effect on the stability of the chlorophyll-protein complexes to temperature-induced dissociation [21]. Knockdown of a chloroplast PGPP1 reduced plastid PG (34:4) and the other 34:x molecular species of PG without affecting the molecular species of PG 32:0 and 32:1 [16]. This mutant shifted glycolipid synthesis towards chloroplast-derived species of DGDG 34:3 and reduced DGDG 36:6 which is derived from ER-assembled precursors, though levels of PC, PE, and PI were not affected [16]. The *pgpp1-1* mutant plants showed reduced chlorophyll content, which did not affect quantum yield [16]. Interestingly, the thylakoid-associated redox protein PRXQ is required for FAD4 activity [17]. The *prxq* mutant exhibited a reduction in 16:1t-PG [22] and increased sensitivity to oxidants [23]. Thus, 16:1t-PG is proposed to be involved in sensing of the redox status of chloroplasts [23].

Since 16:1t-PG is found throughout the plant kingdom, synthesized in green tissue, and associated with photosynthetic machinery, it has been proposed that 16:1t-PG has a fundamental role in eukaryotic photosynthesis [24]. However, complete abolishment of 16:1t-PG does not affect photosynthetic antenna function under standard growth conditions [25]. Genetic dissection of the lipid bilayer composition provides essential in vivo evidence for the role of individual lipid species in membrane function [14]. Lipidomics are increasingly applied to study pathway perturbations in various settings that implicate dysregulation in lipid metabolism [26,27], leading to a novel understanding of the connections between lipids and phenotypes [26,28,29,30]. To offer additional insights about the in vivo role of the *FAD4* gene and 16:1t-PG, we generated *FAD4*-overexpressing plants (*OX-FAD4s*). We measured their growth rates, and quantitated polar lipids changes by lipidomic profiling. Our data suggest that FAD4 is involved in the synthesis of PG (36:7); in addition, 16:1t-PG homeostasis in plastid envelope membranes could regulate plant growth by effecting photoassimilate export from the plastid.

## 2. Materials and Methods

### 2.1. Plant Growth

There are three *FAD4* knockout lines (*fad4-1, fad4-2*, and *fad4-3*) reported before [15]. FAD4 protein possesses three histidine motifs (170QGHH173, 229HAWAH233, and 258HAEHH262) which could be responsible for the activity of membrane-bound desaturases. Thus, for a complete knockout of FAD4 activity, it is ideal to delete all the three histidine motifs. The *fad4-1* presumably produce a truncated protein of 177 amino acids which still retain the first histidine motif; the T-DNA in *fad4-2* (SAIL_1250_C12) was inserted in amino acid residue 132 of the inferred amino acid sequence; while the T-DNA in *fad4-3* mutant was inserted in amino acid residue 287 in which all three histidine motifs were retained. We reasoned that the *fad4-2* mutant is the only line that all histidine motifs are removed; thus, it was selected in this study. Plants were grown in a composite of peat soil: vermiculite: perlite (8:1:1, *v/v/v*); temperature 22 °C; light intensity 160 µmol photon m^−2^ s^−1^; humidity 60%.

### 2.2. Overexpression of the FAD4 Gene in Arabidopsis

*FAD4* CDS was amplified from *Col* total RNA by RT-PCR using a pair of gene-specific primers, with *Bam* HI and *Xho* I recognition sequences added into the forward and reverse primers, respectively. The primer sequences were: 5′-CGGGATCCATGGCTGTATCACTTCC-3′ and 5′-CCGCTCGAGTTATGCTTGGTTGTTGG-3′. The amplified PCR fragment was digested by *Bam* HI and *Xho* I, then was cloned into the pMGmubi vector, in which the *FAD4* gene was driven by a soybean ubiquitin promoter. The plasmid construct was introduced into *Agrobacterium tumefaciens* strain GV3101, then transformed into *Col* by the floral dip method. T_1_ seeds were surface sterilized and germinated on 1x MS plates containing 50 μg mL^−1^ kanamycin. Kanamycin-resistant seedlings were transferred into soil and allowed to self-fertilize. T_2_ seeds were harvested from individual plants, then germinated on 1x MS plates containing 50 μg mL^−1^ kanamycin, six resistant seedlings per line were transferred into soil and self-fertilized, T_3_ seeds were harvested individually. To identify the homozygote transgenic lines, T_3_ seeds were sterilized and germinated on 1x MS plates containing 50 μg mL^−1^ kanamycin, the lines that show uniform kanamycin resistance were judged as homozygote, then used for physiological and biochemical analysis.

### 2.3. RT-qPCR

*Col, fad4-2* and *OX-FAD4s* seeds were surface sterilized and sawed onto 0.5x MS plates, and grown under continuous white light for 17 days, seedlings were harvested, and total RNA was isolated using an M5 Quickspin plant RNA extraction kit with in-column DNase I digestion (Mei5bio, Beijing, China), RNA integrity was checked with 0.7% agarose gel. A total of 2 μg of total RNA was reverse transcribed using M-MLV (Mei5bio, Beijing, China) and random N9 primer in a total volume of 20 μL, then diluted one-fold before being used as a qPCR template. qPCR was performed using a SYBR Premix Es Taq (with Tli RNaseH) (Mei5bio, Beijing, China) and CFX96 Touch module (Bio-Rad, Pleasanton, CA, USA). The PCR condition was: 95 °C, 30 s; 95 ℃, 5 s, 60 °C, 30s, 39 cycles; 95 °C, 10 s, 65 °C, 31 s; 65 °C, 5 s, + 0.5 °C/cycle, ramp 0.5 °C/s, 60 cycles. The *FAD4* primer sequences are: F1: 5′- AGCAAGCTTCCACCTCTCGT-3′; F2: 5′- TCCCTCGCTTCTCCGTGTAC-3′; R1: 5′- TGCTCCACTCACGATGCAGT-3′. *UBQ5* was used as reference gene. Each reaction was performed in triplicate along with internal control reaction. Relative gene expression levels were calculated according to the 2^−∆∆Ct^ comparative CT method.

### 2.4. Plant Growth Parameter Measurement

Seeds of *Col, fad4-2, OX-FAD4_2* and *OX-FAD4_5* were surface sterilized and sawed onto 1x MS plates, kept in 4 °C freezer for 2 days, then moved into continuous white light for 9 days. The seedlings were transplanted into soil and grown under continuous light for an additional 15 days before photography. The projected leaf area was calculated by using Image J software. The number of leaves were counted from the individual plant. The above-ground rosette leaves from individual plants were harvested and weighed by a balance (Jing-Tian, Shanghai, China) to obtain a fresh weight, then dried overnight in a 105 °C oven to obtain the dry plant weight.

### 2.5. Total Lipid Extraction

*Col*, *fad4-2*, and *OX-FAD4_2* plants were grown in soil under continuous light for three weeks, above-ground tissues were harvested, total lipids were immediately extracted using the single step extraction method, as described previously [31], five biological replicates were prepared. This extraction method divides plant mass into lipid fraction and insoluble residue. The lipid fraction was completely dried under a gentle nitrogen stream to obtain dry lipid weights, then stored at −80 °C before lipidomic profiling; the insoluble residue was dried overnight in a 105 ℃ oven to obtain dry residue weight. Total plant dry mass = dry lipid weight + dry residue weight.

### 2.6. Lipidomic Profiling

Phospholipid and galactolipid internal standards were used for individual lipid class identification and quantification [32]. At least two different lipid species per lipid class were used. Internal standards and their acquisition information are provided in Table S1. To prepare the samples for MS analysis, for each sample, the volume corresponding to 7.5 to 9.6 μg dried lipid was transferred to a vial containing the internal standards indicated in Table S1. Samples were brought to 1 mL by adding chloroform: methanol: 300 mM ammonium acetate in water (30/66.5/3.5, *v/v/v*) for mass spectrometric analysis.

The samples were analyzed on an electrospray ionization tandem mass spectrometer (ESI-MS/MS) (Waters Xevo TQS mass spectrometer; Waters Corporation, Milford, MA, USA) using sequential precursor and neutral loss scans (Table S2) and processed, as described previously [33]. The source temperature was 150 °C, desolvation temperature was 250 °C, cone gas flow was 150 L/h, collision gas (argon) flow was 0.14 mL/min, nebulizer gas was at 7 Bar, the LM 1 resolution was set at 3.2, and the HM resolution was set at 15.5. With the analytical platform used (Waters Xevo TQS), the intensity observed for galactolipid species varies in relation to the number of double bonds in the acyl chains. The data were corrected for the response variation using the response factors indicated in Table S3.

### 2.7. Statistical Analysis

Average and standard errors were calculated in Excel software (Microsoft, Seattle, WA, USA). One-way ANOVA was used to determine the significance based on Duncan’s multiple range tests in SPSS software (V22; IBM, Armonk, NY, USA).

## 3. Results

### 3.1. Confirmation of FAD4 Overexpression and Knockout lines by RT-qPCR

**Figure 2:**
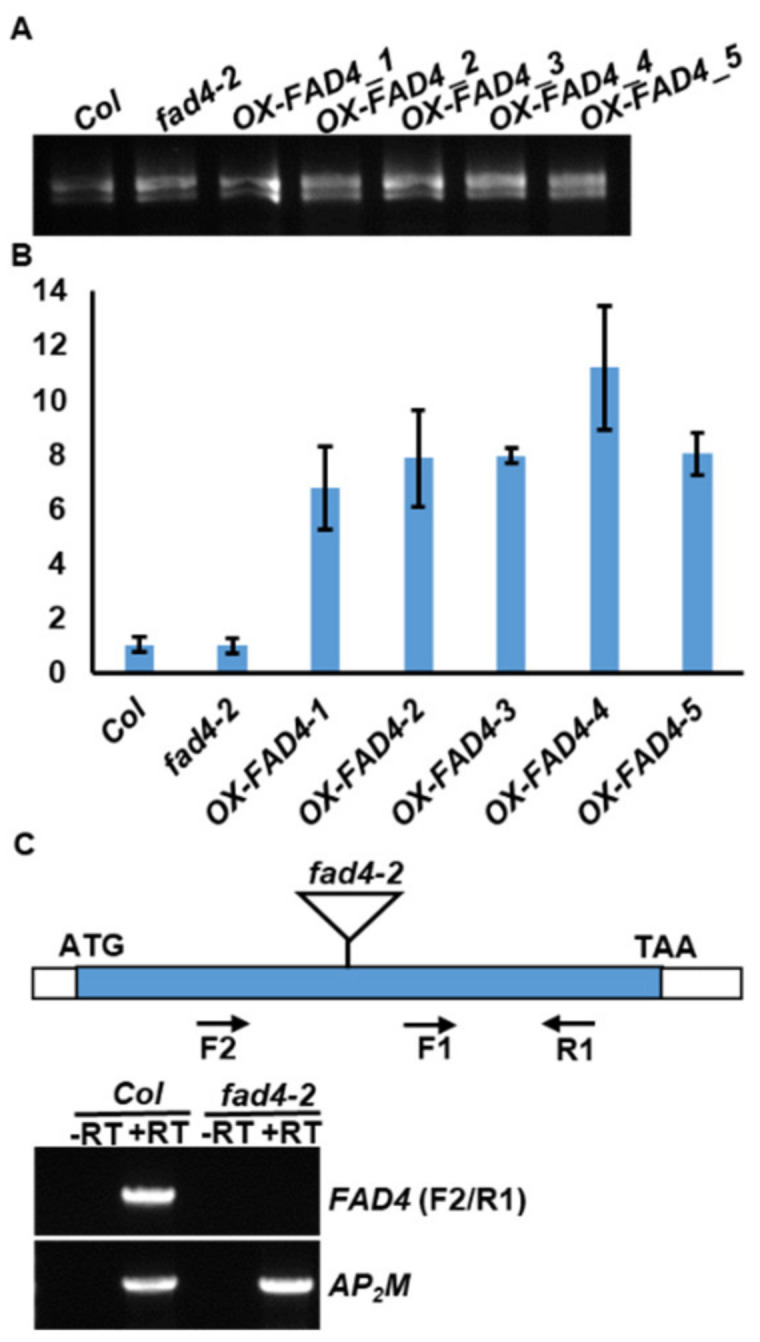
Overexpression or knockout of *FAD4* in *Arabidopsis*. (**A**) Agarose electrophoresis of total RNA isolated from *Col, fad4-2* and *OX-FAD4s* plants. (**B**) *FAD4* expression levels measured by RT-qPCR. (**C**) T-DNA insertion in *fad4-2* produces a long *FAD4* chimera mRNA that may translate into a nonfunctional FAD4 protein. –RT: reverse transcription without M-MLV addition as negative control; +RT: reverse transcription with M-MLV addition. AP_2_M (At5g46630) was used as loading control.

Gao et al. (2009) demonstrated that the FAD4 enzyme introduces a Δ^3-trans^ double bond to palmitic acid esterified at the sn-2 position of PG [15] and is the key enzyme for PG (34:4) (PG18:3/16:1t) synthesis through the prokaryotic pathway. To offer additional insights about the functions of FAD4 and its products, we overexpressed the *FAD4* CDS sequence driven by a soybean ubiquitin promoter in the *Col* background. To confirm whether transgenic *FAD4* was properly expressed, total RNA was isolated (Figure 2A) and reverse transcribed, then RT-qPCR was conducted. The *FAD4* expression levels in *OX-FAD4* lines were 7–10 fold higher than that of the WT control (Figure 2B). Surprisingly, the *FAD4* level in *fad4-2* knockout plants was similar to the WT control when F1/R1 primer sets were used for RT-qPCR (Figure 2B,C). To test whether T-DNA insertion in *fad4-2* affects its *FAD4* mRNA processing, the primer set F2/R1, which flanks the T-DNA insertion site, was used for regular RT-PCR amplification (Figure 2C). The F2/R1 primer set specifically amplified a fragment with an expected size from the WT control but failed from the *fad4-2* sample (Figure 2C). These data suggest that the T-DNA insertion in *fad4-2* produces a long *FAD4* chimera mRNA, which may translate into a defective FAD4 protein. These data are in accordance with Gao et al. (2009) who reported that *fad4-2* showed similar defects on the PG profile as other *fad4* mutant alleles [15].

### 3.2. Overexpression or Knockout of FAD4 Enhances Plant Growth

**Figure 3:**
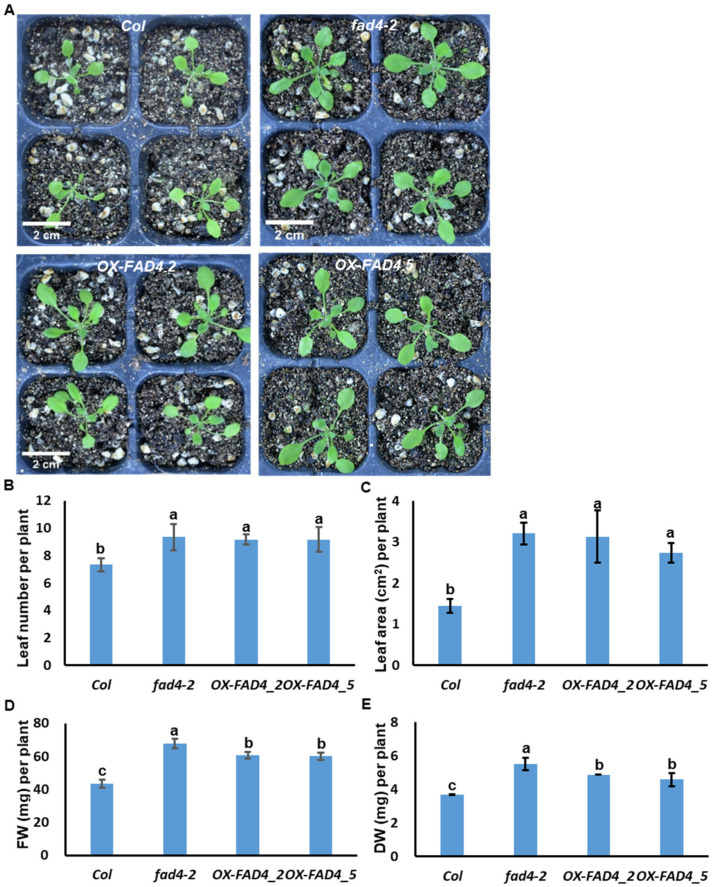
Overexpression or knockout of *FAD4* in *Arabidopsis* enhanced plant growth under continuous light. (**A**) Photography of seedlings after grown 24 days under continuous light. (**B**–**E**) Average leaf number, average projected leaf area, average fresh weight, and average dry weight per plant of *Col*, *fad4-2*, *OX-FAD4_2* and *OX-FAD4_5*. Data were expressed as average ± standard deviation (n = 6). Different letters indicate significant differences at *p* < 0.05 by one-way ANOVA with Duncan’s multiple range test. Bar = 2 cm in (**A**).

To characterize whether *FAD4* expression levels affect plant growth, WT, *fad4-2*, and two *OX-FAD4s* (*OX-FAD4_2* and *OX-FAD4_5*) were grown under continuous white light condition. *OX-FAD4_2* and *OX-FAD4_5* showed similar expression levels of *FAD4* (Figure 2B); thus, were chosen for phenotypic characterization in order to minimize the potential gene dosage effects on phenotypic variations. *Col* plants were visibly smaller compared to *fad4-2* or *OX-FAD4s* (Figure 3A). Physiological parameters, including leaf number per plant, projected leaf area, fresh plant weight, and dry plant weight, were measured. At same stage, *fad4-2* and *OX-FAD4s* plants had more leaves (Figure 3B), suggesting that the disruption of *FAD4* expression enhanced plant vegetative growth rates. The projected leaf area, fresh weight, and dry weight of *fad4-2* and *OX-FAD4s* also were higher than the WT control (Figure 3C–E).

### 3.3. Disrupting FAD4 Homeostasis Reduced Total Lipid Content but Increased Polar Lipid Proportion

**Figure 4:**
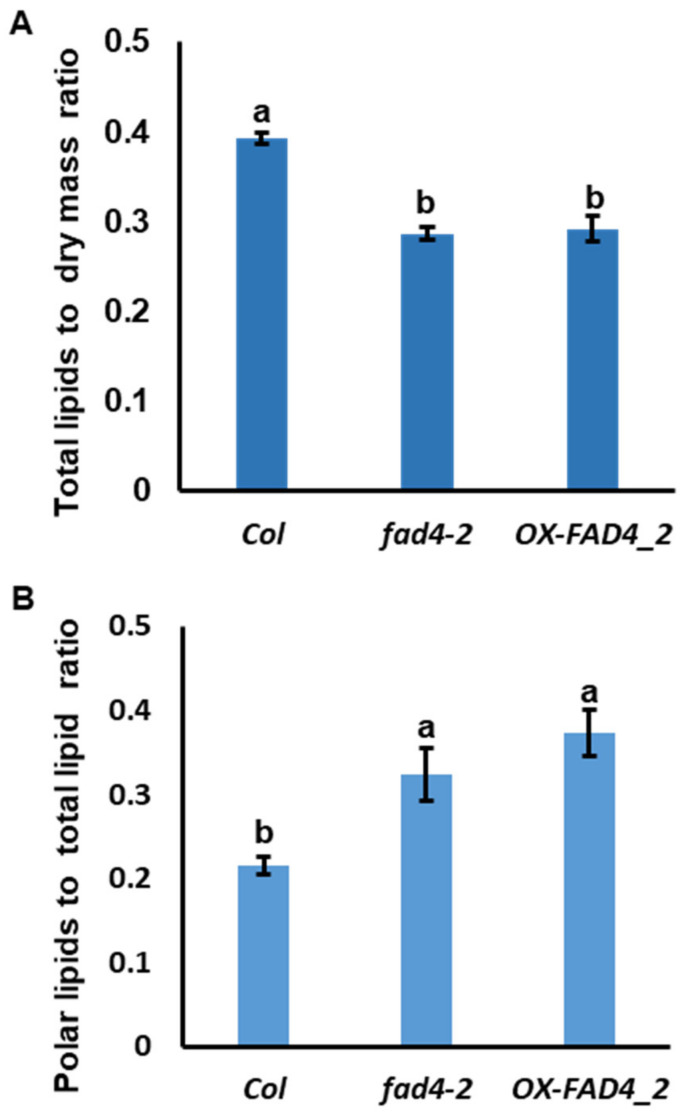
Disruption of *FAD4* expression altered the ratio of total lipids to dry plant mass and the ratio of polar lipids to total lipids. (**A**). Total lipids to dry plant mass ratios of *Col*, *fad4-2*, and *OX-FAD4_2* plants; (**B**). The polar lipids to total lipid ratios of *Col*, *fad4-2*, and *OX-FAD4_2* plants. The difference letter represent statistically significant.3e.

To dissect the functions of *FAD4* for 16:1t-PG and other potential lipid synthesis, we decided to choose *fad4-2* as one representative *FAD4* knockout line, and *OX-FAD4_2* as one representative *FAD4* overexpression line. We reasoned that through a small scale multiple parallel comparison of lipid changes among WT, *fad4-2* and *OX-FAD4_2*, we might be able to validate its established functions and discover novel roles. Thus, the total lipids were extracted from *Col*, *fad4-2*, and *OX-FAD4_2* by the single step extraction method, as described previously [31]. The total lipid to dry plant mass ratios from *fad4-2* and *OX-FAD4_2* were reduced by ~25% compared to the WT control (Figure 4A). In WT, this ratio was ~0.4, which is higher than expected for plant vegetative tissue. Considering that the solvent used for lipid extraction contains chloroform: isopropanol: methanol: water (22.5/25/31.1/2.6, *v/v/v/v*), we speculate that most polar metabolites could also be extracted into the so-called “total lipids”. Under such a scenario, the residue left after lipid extraction mainly represents insoluble plant materials such as cell wall components (cellulose, semicellulose, and lignin, etc.). Multiple factors could be accountable for the reduced ratio in *fad4-2* and *OX-FAD4* plants, including reduced polar metabolite, elevated cellulose or lignin synthesis, reduced polar lipid or non-polar lipid content. Additionally, this could be due to developmental differences between mutant and wild type.

Polar lipids from *Col*, *fad4-2*, and *OX-FAD4_2* were analyzed by lipidomic profiling of 157 polar lipid species, the ratio of total polar lipid to the total lipid dry mass were calculated. We found that the polar lipid proportions in *fad4-2* and *OX-FAD4_2* were 50% and 73% higher than that of WT control (Figure 4B), indicating that polar lipid synthesis in *fad4-2* and *OX-FAD4_2* were not decreased.

### 3.4. Lipidomic Profiling Confirmed FAD4 Enzyme Activities for Prokaryotic PG Synthesis

To compare polar lipid changes among WT, *fad4-2*, and *OX-FAD4_2*, each polar lipid species was normalized to plant dry weight and data expressed as nmol mg^−1^ plant dry weight (Table S4). In addition, polar lipid values were transformed into mol% by normalizing to the total polar lipid content of the sample, which represents the overall polar lipid composition of the plant cell (Table S5).

**Figure 5:**
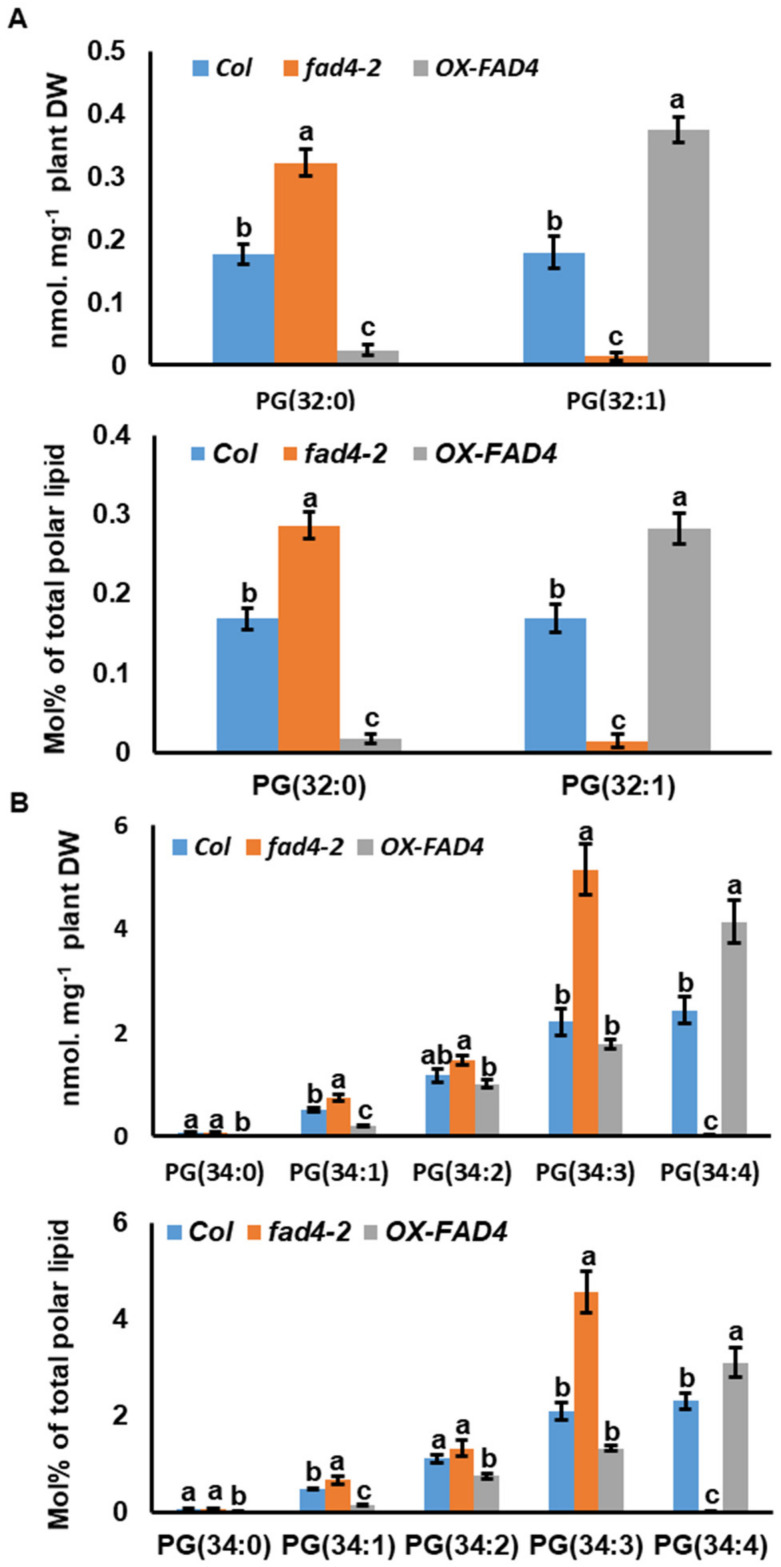
PG (32) and PG (34) contents and Mol % distribution from total polar lipids of WT, *fad4-2*, and *OX-FAD4_2* plants. (**A**) PG (32) contents (upper panel) and Mol % distribution from total polar lipids (lower panel). (**B**) PG (34) contents (upper panel) and Mol % distribution from total polar lipids (lower panel). Data were expressed as mean ± standard error (n = 5). Different letters within the same molecular species represent statistically significant difference.

PG species were presented as PG (X:Y), where X represents the total carbon number and Y represents the total double bond number. In total, fourteen PG species were detected: PG (32:0), PG (32:1), PG (34:0), PG (34:1), PG (34:2), PG (34:3), PG (34:4), PG (36:1), PG (36:2), PG (36:3), PG (36:4), PG (36:5), PG (36:6), and PG (36:7) (Table S4). In *Arabidopsis*, PG (32) has two 16C fatty acids esterified at the *sn*-1 and *sn*-2 positions of the glycerol backbone, respectively. Hsu et al. (2007) demonstrated that the double bond present in PG (32:1) is in the *trans*-configuration [34]. Thus, PG (32:1) can be written as PG 16:0/16:1t. In WT, PG (32:0) (PG16:0/16:0) and PG (32:1) were detected at almost equal amounts in terms of nmol mg^−1^ total plant dry weight. Compared to the WT control, PG (32:1) content in the *fad4-2* mutant was reduced 92%, and PG (32:0) content increased 83% (Figure 5A). In contrast, the PG (32:1) content in *OX-FAD4_2* increased 2.1-fold, and PG (32:0) content decreased 86% compared with WT control (Figure 5A, upper panel). When the data are expressed as mol%, the changing trends and significance were not affected (Figure 5A, lower panel; Table S5).

PG (34) species can be derived either from the prokaryotic pathway with 16C FA esterified at the *sn*-2 position (PG 18:X\16:1t, X represents the number of double bonds), or from the eukaryotic pathway with 18C FA esterified at the *sn*-2 position (PG 16:0\18:X), since eukaryotic PG usually has a saturated palmitic acid at the *sn*-1 position. Thus, PG (34:4) is almost exclusively derived from the prokaryotic pathway (PG 18:3\16:1t). When the data were expressed as nmol mg^−1^ total plant dry weight, compared to WT control, PG (34:4) content from the *fad4-2* mutant plants decreased 99%, and the amount of PG (34:1), PG (34:2), and PG (34:3) increased 44%, 25%, and133%, respectively (Figure 4B, upper panel). In contrast, compared to the WT control, PG (34:4) content from the *OX-FAD4_2* plants was 70% higher; PG (34:0) and PG (34:1) contents were 87% and 62% lower; while PG (34:2) and PG (34:3) contents in *OX-FAD4_2* were not significantly affected (Figure 5B, upper panel). When the data were expressed as mol%, compared to WT control, the proportion of PG (34:4) from the *fad4-2* mutant plants decreased 99%, while the proportion of PG (34:3) increased 119%. In contrast, the proportion of PG (34:4) from the *OX-FAD4_2* plant increased 35%, PG (34:0), PG (34:1) and PG (34:2) all significantly decreased (Figure 5B, lower panel).

### 3.5. Lipidomic Profiling Uncovered a Potential New Substrate for FAD4

**Figure 6:**
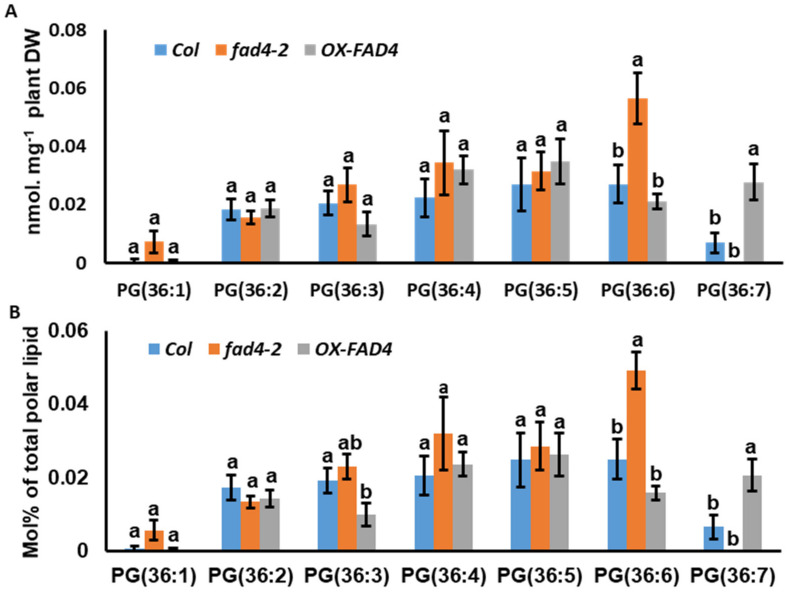
PG (36) contents and Mol % distribution from total polar lipids of WT, *fad42*, and *OXFAD4_2* plants. (**A**) PG (36) contents from WT, *fad42*, and *OXFAD4_2*. (**B**) PG (36) Mol % distribution from total polar lipids of WT, *fad42*, and *OXFAD4_2*. Data were expressed as mean ± standard error (n = 5). Different letters within same molecular species represent statistically significant difference.

PG in extraplastidic membranes is nearly exclusively of eukaryotic structure with 18C fatty acids at the *sn*-2 position [35,36]. PG 34 (16/18) is the dominant PG in plasma membranes [36,37], while trace amounts of PG 36 (18/18) have been reported [35]. Fritz et al. (2007) and Burgo et al. (2011) found Δ^3-trans^ in *sn*-2 bound *cis*-unsaturated 18C fatty acids of PG [38,39], which raises the question whether FAD4 is responsible for introducing this *trans*-double bond. Thus, we took a close look at the PG (36) fraction. Consistent with a previous report [35], our lipidomic data showed that PG (36) lipids were much lower compared with the PG (34) lipids (Table S4). In WT, PG (36:7) was detected in 3 out of 5 biological replicates but was non-detectable in all five biological replicates of the *fad4-2* mutants. This may explain why there were no statistically significant differences in PG (36:7) content between WT and the *fad4-2* mutant plants (Figure 6A). Accordingly, the PG (36:6) content in *fad4-2* increased 2.1 fold compared with the WT control, while other PG36 species (36:1, 36:2, 36:3, 36:4, and 36:5) were not significantly affected. In contrast, PG (36:7) was detected from all five biological replicates of *OX-FAD4_2* plants and 3.0 folds higher than that of WT, while the other PG (36) molecular species were not significantly affected (Figure 6A; Table S4). When the data were expressed as mol%, PG (36:7) from *OX-FAD4_2* plants was 2.1 folds higher than that of WT, while the other PG (36) species were not affected (Figure 6B). In the *fad4-2* mutant, the proportion of PG (36:6) increased 96% (Figure 6B). Our data are consistent with the notion that FAD4 may catalyze PG (36:7) synthesis [38,39].

### 3.6. Disruption of FAD4 Expression Reduced LysoPG Contents

**Figure 7:**
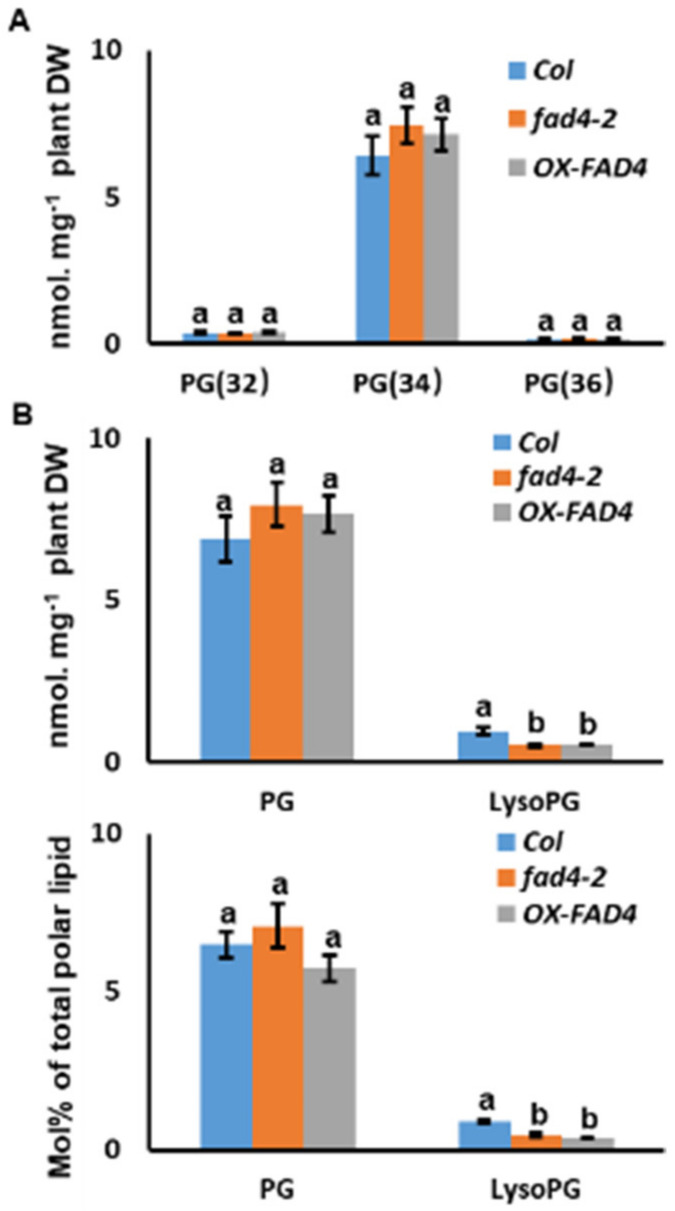
PG and LysoPG contents and Mol % distribution from total polar lipids of WT, *fad42*, and *OXFAD4* plants. (**A**). The pool size of PG(32), PG(34), and PG(36) of WT, *fad42*, and *OXFAD4_2* plants. (**B**). The PG and lysoPG contents (upper panel) and Mol% distribution from total polar lipids (lower panel) of WT, *fad4-2*, and *OX*-*FAD4_2* plants. Different letters within same chemical class represent statistically significant difference.

Even though individual molecular species of PG (32), PG (34) and PG (36) from *fad4-2* and *OX-FAD4_2* showed predictable changes compared to WT plants (Figure 5 and Figure 6), the total subpool sizes of PG (32), PG (34), PG (36) did not show any significant differences (Figure 7A). The total PG pool sizes as well as Mol% distributions were also similar among *WT*, *fad4-2* and *OX-FAD4_2* plants (Figure 7B, upper panel). However, lysoPG pool sizes in *fad4-2* and *OX-FAD4_2* were consistently lower than that of WT regardless of the normalization method (Figure 7B, lower panel).

### 3.7. Over-expression of FAD4 Slightly Enhanced Prokaryotic and Eukaryotic MGDG Synthesis

**Figure 8:**
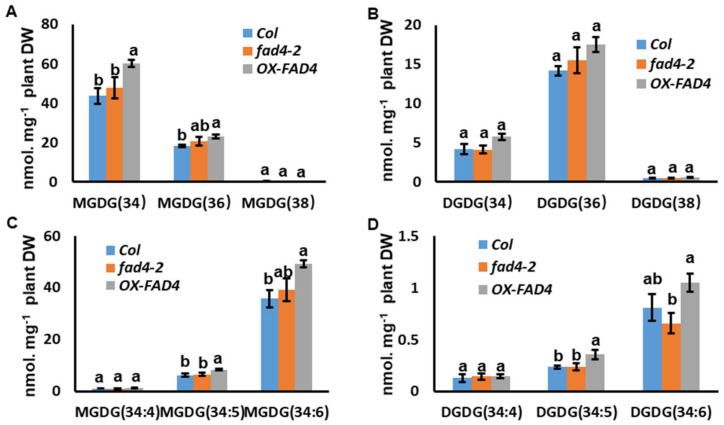
The contents of MGDG and DGDG molecular species from WT, *fad42*, and *OXFAD4_2* plants. (**A**) MGDG molecular species contents from WT, *fad42*, and *OXFAD4_2*. (**B**) MGDG (34) molecular species contents from WT, *fad42*, and *OXFAD4_2*. (**C**) DGDG molecular species contents from WT, *fad42*, and *OXFAD4_2*. (**D**) DGDG (34) molecular species contents from WT, *fad42*, and *OXFAD4_2*. Different letters within the same molecular species represents a statistically significant difference.

*OX-FAD4_2* showed a larger pool size of MGDG (34) and MGDG (36) compared with WT; this increase was not seen from *fad4-2* mutant plants (Figure 8A). The pool size for DGDG (34), DGDG (36) and DGDG (38) all were similar among WT, *fad4-2* and *OX-FAD4_2* (Figure 8B). MGDG (36) is specifically derived from precursors assembled on the ER since its sn-2 position is a 18C fatty acid. The higher contents of MGDG (36) of *OX-FAD4_2* suggests that over expression of *FAD4* stimulates eukaryotic MGDG synthesis.

34:X glycolipids (MGDG and DGDG) can originate from both prokaryotic and eukaryotic pathways. ER-derived “34” DAG-backbones contain 16:0 saturated acyl moieties at the *sn*-1 position of the glycerol backbone [2]. Thus, we can assume that MGDG 34:X and DGDG 34:X, with more than four desaturated C-bonds, are exclusively synthesized by the prokaryotic pathway [3]. Thus, the levels of MGDG 34:4, MGDG 34:5, MGDG 34:6, DGDG 34:4, DGDG 34:5, and DGDG 34:6 are diagnostic. MGDG 34:5 and MGDG (34:6) levels in *OX-FAD4_2* line showed a small but statistically significant increase compared to WT, while *fad4-2* was not affected (Figure 8C). These data suggest that overexpression of *FAD4* also stimulates prokaryotic MGDG synthesis.

### 3.8. Disruption of FAD4 Expression Did Not Affect PC, PE, PI, PS and PA Contents or Compositions

**Figure 9:**
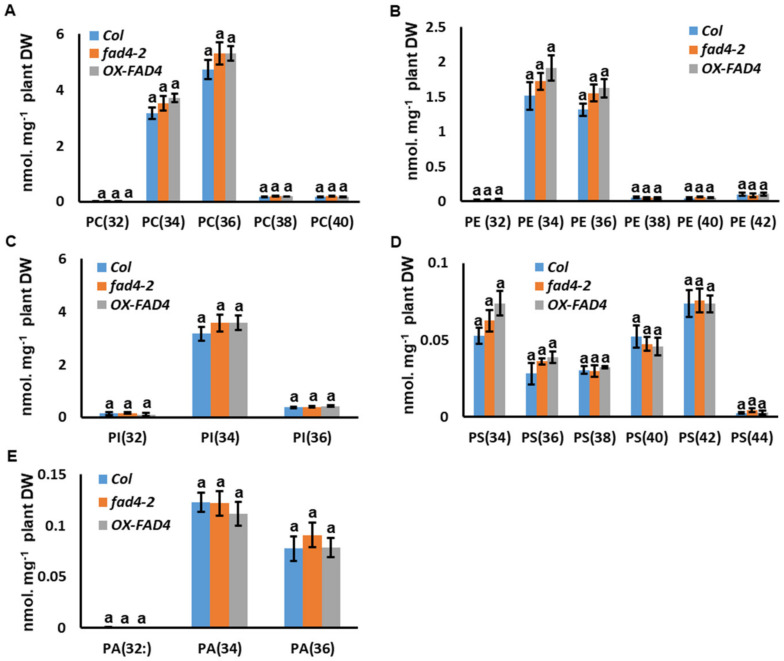
The molecular species contents of PC (**A**), PE (**B**), PI (**C**), PS (**D**), and PA (**E**) from WT, *fad42*, and *OX*-*FAD4_2* plants. Different letters within the same molecular species represent statistically significant differences.

To evaluate the potential roles of FAD4 for other polar lipid metabolism, the contents of PC, PE, PI, PS, and PA were compared among WT, *fad42*, and *OXFAD4_2* plants. Our data demonstrated that these polar lipids were not significantly affected by knockout or overexpression of the *FAD4* gene in *Arabidopsis* (Figure 9).

## 4. Discussion

Lipidomic profiling of WT, *fad4-2*, and *OX-FAD4_2* plants revealed changing patterns of PG lipid species (Figure 4) that are in accordance with previous reports that FAD4 is responsible for the biosynthesis of PG (32:1) and PG (34:4) [15,34,40]. In addition, our data (Figure 6) suggest that FAD4 is involved in PG (36:7) biosynthesis, as also proposed previously [38,39]. Hurlock et al. (2018) demonstrated that ER-derived precursors are imported and contribute to plastid PG synthesis [7,41]. Horn et al. (2020) further showed that recombinant FAD4 can use 16:1^Δ9^ or 18:1^Δ9^ as a substrate and introduce a Δ3 double bond [17]. These findings provide important insights on how PG (36:7) be synthesized. We hypothesize that ER-assembled PC36 (18:1\18:1) is imported into the plastid, possibly through the TGDs complex. At the IE membrane, PG (36) (18:1\18:1) is synthesized via the plastid PGP1- and PGPP1-mediated synthesis pathway. After formation, PG (36) (18:1\18:1) is transported to thylakoids, wherein a trans-double bond is introduced into the Δ3 position of the 18:1 FA at the *sn*-2 position by FAD4, then further desaturated by FAD6/FAD7/FAD8 into PG (36:7) [42,43,44].

Our lipidomic profiling experiment demonstrated that disturbing *FAD4* expression effectively altered PG content and composition (Table S4, Figure 5 and Figure 6). Even though PG (34:4) synthesis was almost completely abolished in the *fad4-2* mutant (Figure 5B) and the PG (34:4) content increased by 70% in the *OX-FAD4_2* plants (Figure 5B), most other polar lipid classes exhibited similar trends between *fad4-2* and *OX-FAD4_2* (Figure 9), suggesting the high substrate specificity of FAD4 enzyme. One exception is prokaryotic and eukaryotic MGDG synthesis, which was differential between *fad4-2* and *OX-FAD4_2* lines (Figure 8D). Considering that the *trans*-double bond containing PGs including PG (32:1), PG (34:4), and PG (36:7) were the major PG lipids up-regulated in *OX-FAD4_2* compared to WT (Figure 5 and Figure 6), we speculate that *trans*-double bond-containing PGs could influence MGD1 or MGD2 activities on the plastid envelope membrane.

Interestingly, we found that knockout or overexpression of *FAD4* led to enhanced plant growth and accompanied with reduced total soluble metabolites in vivo (Figure 4A). These data were in accordance with the previous observation that biomass is negatively correlated with the intermediates of central metabolic pathways [45]. Plastids are the only site for photosynthesis, which provides the sole carbon source to feed plant growth. The photosynthetic end product (3-PGA) exits the plastid stroma to the cytoplasm through TPT translocator, then converts into sucrose and transport into other sink organs. Meanwhile, plastids are also the site for several anabolic pathways, including fatty acids, transit starch, shikimate pathway, aromatic amino acids, and phytohormones (GA, ABA), etc. Since plants function as integrated systems, the distribution of metabolites between growth, and production of defense and storage compounds has to be tightly regulated [45]. It is speculated that photoassimilate export could be the first check point to balance plant growth and defense. Previous research demonstrated that plant growth is limited to a submaximum level to enable plants to cope with unfavorable conditions [46]. To support this, 16:1t-PG has been associated with stress response. For example, thylakoid membrane-associated *PRXQ* mutation led to ~75% reduction of 16:1t-PG [17]. Accordingly, the Arabidopsisi *prxq* mutant plants showed increased sensitivity to oxidants [23]; in copea, a cold-sensitive plants, 16:1t-PG was negatively associated with the robustness of photosynthesis and contributed to chilling sensitivity [47]. These studies demonstrated that 16:1t-PG plays important roles in coordinating plant metabolism and stress responses. Our data suggest that 16:1t- PG on plastid membranes could regulate the export of photoassimilate or other metabolites from chloroplast that affect plant growth. Although the underlying mechanisms still await further characterization, here we propose two potential routs for 16:1t-PG to fulfill such role: 1) plastid 16:1t-PG homeostasis could be a critical factor to determine membrane permeability coefficients for metabolites; 2) plastid 16:1t-PG could interact with plastidic translocators or membrane associated proteins, including the recently discovered CTI family of envelope membrane proteins, which directly affect acetyl-CoA carboxylase activity [48]. Disruption of PG homeostasis on plastid membranes could lead to accelerated photoassimilate export; thus, altering sink/source ratios and enhancing plant growth [49]. In return, the enhanced plant growth draws upon photoassimilate leading to lower apparent soluble metabolite levels (Figure 3 and Figure 4A).

## 5. Conclusions

In this study, we generated *FAD4* overexpression plants (*OX-FAD4s*), then evaluated the growth performance among WT, *fad4-2* knockout mutant, and *OX-FAD4_2* plants. We showed that knockout or overexpression of *FAD4* led to enhanced plant growth, the total extractable soluble metabolite contents were negatively correlated with their enhanced growth. Lipidomic profiling of polar lipids showed FAD4 is involved in PG 32:1, PG 34:4 and PG 36:7 synthesis; while prokaryotic and eukaryotic MGDG was up-regulated only from *OX-FAD4* plants but not *fad4-2* knockout plants. This study provides novel insights about the roles of FAD4 on plastid PG homeostasis, plant growth and metabolism.

## Data Availability

The data presented in this study are available in article and Supplementary Materials.

## Supplementary Materials

The following supporting information can be downloaded at: https://www.mdpi.com/article/10.3390/metabo13030318/s1, Table S1: Phospholipid and galactolipid standards and amounts used for individual lipid class identification and quantification; Table S2: ESI-MS/MS method parameters; Table S3: The response factor for individual glycolipids relative to saturated MGDG or DGDG standards; Table S4: Col, fad4-2, and OX-FAD4 lipidomic profiling dataset expressed as nmol. mg−1 plant dry weight; Table S5: Col, fad4-2, and *OX-FAD4* lipidomic profiling dataset expressed as mol%.
